# Dr. Clarke, on Vaccination

**Published:** 1806-05

**Authors:** James Clarke

**Affiliations:** Physician to the Vaccine Institution. Nottingham


### *To the Editors of the Medical and Physical Journal.*

Gentlemen,

PERMIT me, through the medium of the Medical and Physical Journal, to make some observations on a paper in your last Number, denominated, "Extracts from the Proceedings of the Institution to promote Cow-pox Inoculation in the town of Nottingham," but more particularly on the latter part of that paper, *extracted from a letter addressed to the Secretary of the Royal Jennerian Society*; without the most remote idea of appearing before the tribunal of the public.

Although

Although a long extract has been made from the Proceedings of this Institution, yet, what may be considered as a very material part, the Regulations, are omitted; certainly the machinery, by which the movements are directed: they are extremely simple, and as such more valuable: I shall claim your permission to insert them.

"At a meeting of the Directors of the Institution for Vaccine Inoculation, held for the first time on the 31st of October, 1805, it was agreed, that for the better attainment of the purposes of the Institution, and the better regulation of their proceedings, the following general Rules be observed.

"1. That the Directors shall meet twice at least in every month, for the purpose of receiving Reports of the state of Vaccination, of inspecting the records, and adopting such measures as shall appear to them to be the best calculated to promote the success of that practice.

"2. That the Directors of this Institution do not propose to take upon themselves any share in the executive part of this plan, except that the medical members of it shall at all times be ready, when called upon, to give their advice, either individually or in consultation, on any question of doubt or difficulty that may arise in the practice.

"3. That a Physician be appointed to the Vaccine Institution, and that it shall be his duty to be ready to give his advice, when called upon by the surgeon; when any doubt arises respecting the propriety of inoculating any individual, or in any case of irregular symptoms occurring during the process, to see each patient that is vaccinated under the direction of the Institution, at least, once during the process; and, that more especially in its advanced stage, that he may be prepared to give his opinion how far it has proved satisfactory or otherwise; and to request, whenever he may judge it necessary, the assistance of any one or more of the medical members of the Institution, to decide any question of doubt or difficulty that may arise.

"4. That a Surgeon be appointed to this Institution, whose duty it shall be to visit all the poorer families of the three parishes in the town, in regular rotation, beginning at some one part of it; to encourage and persuade them to accept the advantages offered by the Institution; and to vaccinate as many as are willing and are deemed fit subjects for this practice; to visit each patient that has been vaccinated, at three distinct periods after vaccination, and make as many written Reports on the case, adding such observations on each case as shall be thought necessary by the physician and himself.

"In case of any irregularity occurring in the progress of the symptoms, to call upon the physician for his assistance, and to keep a regular register of each case in a tabular form, including the names, age, and dwelling of each patient, and three distinct Reports of the progress and observations as above stated."

These Resolutions of the Directors require little comment; under their benign influence 903 persons have been inoculated.

The Public Register of the Institution has twelve columns, a blank page for remarks, and a case-book for those of more importance; such trouble may be considered superfluous, yet that labour is not to be regretted which puts us in possession of such accuracy, as to defy calumny when or where it may arise.

The poor are vaccinated at their own habitations:—without such an arrangement this charitable Institution would be almost nugatory. The surgeons here had in the most laudable manner devoted two hours once or twice a week to gratuitous inoculation; this was found very inadequate to their benevolent views; few can give up that time which is so necessary to an attendance on the surgeon; this neglect prevented that regular inspection, which is indispensable to ascertain the progress of the disease; and from conviction of the inefficacy of that plan, an Institution was established.

Of the Address to the Inhabitants of Nottingham, a part only is inserted; I have no doubt it was observed, that a similar statement had been made to the public; a repetition was certainly unnecessary in a publication of such extensive circulation. But its efficacy here, among the lower class, to whom above 4,000 copies have been distributed, is beyond our expectations; it appears to carry conviction to their minds, and few, very few, hesitate to receive the blessing *Jenner* has so liberally bestowed on mankind.

I shall proceed to make some observations on the Extracts from my Letter, and on the Notes of Dr. Walker; but first let me say, that I shall be flattered by any communications from your ingenious Correspondents.

In page 255, line 5, "The progress of the disease," I have said, "was soon observed to be much retarded, either by very cold weather, or by much exposure to cold, particularly particularly in debilitated children, who form the majority of the poor of this place."

Dr. Walker's Note does not offer any explanation, but an additional and well known cause of this retarded state.

Page 255, line 26, " Each patient is visited four times; on the third visit, which is generally on the tenth day, a purgative of jalap, or jalap and calomel in combination, is prescribed; we were induced to adopt this plan in compliance with popular prejudice;" and Dr. W. should have added, " but as the children of the poor are generally of very gross habits, we have often found the fever and erythema moderated, as well as the child improved in its general health;" and I may now say, that farther experience has proved to us its great utility.—Dr. W's commentary on this passage is only referable to the practice of the Royal Jennerian Society.

Page 255, line 33, " But when from the test and one or more inoculations no effect is produced, they are considered unsusceptible of cow-pox."—Dr. Walker's note is extremely just; when we are obliged to reinoculate, two or three punctures are made in each arm, inserting fresh fluid ichor; even if we do not then succeed, they are only marked as at that time insusceptible of cow-pox; and if they have not been formerly much exposed to small-pox, so as to render it doubtful if they have not had that disease, or what the poor call the small-pox fever, (which is fever during an exposure to the disease without any perceptible eruption), they are reinoculated at a future period; for this necessary perseverance we are indebted to Dr. Walker.*

The distinction between vesicle and pustule, page 256, line 1, is a subject of much importance, and leads to many practical conclusions: In the usual progress of the disease it is a *vesicle* before the appearance of the erythema and induration; † the fluid will then become *puriform,* which gradually concreting forms a *scab* of a dark mahogany colour.

It has been recommended when the *vesicle* is ruptured to stop the the farther discharge by applying to it a drop of the aq. lythar. acet.‡ this, when used undiluted, proves

* Vide Medical and Physical Journal, vol. 12, p. 45.

† Willan, on Cutaneous Diseases, Def. 10th and 12th.

‡ Address of the Royal Jennerian Society, p. 49. Bryce on Cow-pox, p. 226.

a powerful escharotic; but will answer the purpose when mixed with water. A troublesome ulcer has in some cases followed the rupture of a *pustule*, which we are advised to bathe with the aq. lytharg. acet. comp.* I am inclined to suspect that this application, and the constant exposure of the naked arm, may, in some constitutions, by suddenly stopping the discharge, lay the foundation for an absorption, which produces great induration and enlargement of the axillary glands, which sometimes suppurates, but generally heals favourably.

The only method we have found to prevent this absorption is by the application of the unguent. spermat. ceti to the ruptured pustule, and treating it in every respect as a simple ulcer; but when absorption to any extent takes place, in conjunction with this application a brisk cathartic generally proves effectual.

The Note at the bottom of page 256, demands most serious consideration. "In the number which it continually falls to my lot to inoculate," says Dr. W. "it is generally extremely easy to determine by the eye when the effect is complete; but, when the pock has been ruptured, and even almost obliterated, if I can feel, about the tenth day, a degree of hardness and tumefaction about the inoculated part, I find in people of every colour that the protection is complete; and without such inflammation (induration?) I suspect that the effect is never complete."—This is an opinion entirely new to me, presenting a perfect diagnosis between the true and what has been called the spurious cow-pox; and if I understand the Note, the appearances before or after the tenth day are of little importance, so that on the tenth day a degree of hardness and tumefaction is felt about the inoculated part: I confess I may interpret Dr. W's language too literally.

Mr. Drew relates two cases,† in which induration, with the other usual marks of the true disease, regularly appeared; from which Dr. Walker, with others of great experience in vaccination, pronounced the *protection complete*; yet both these cases afterwards had the small-pox.

Not taking this opinion to the extent mentioned, if it be only applicable when a *pustule* is ruptured, the *vesicle* having proceeded regularly, we are under much obligation

* Address of the Royal Jennerian Society, p. 50. Bryce on Cow-pox, p. 231.

† Medical and Physical Journal, vol. 14, p. 537.

tion to Dr. W.; for nothing in vaccination has given us so much trouble as this state of uncertainty arising from ulceration. We shall speedily bring it to the only certain test,—Experiment.

Page 256, line 7, "We have not found any inconvenience to attend inoculation during teething, hooping cough, &c. provided that the fever of these affections be not violent."—The hooping cough has lately been very virulent, and I believe in every case where the disease was present at inoculation, the vesicle assumed the peculiar flat surface as before related, the pustule and scab presented the same shape and appearance, but in every other respect was as usual. In two or three cases where the hooping cough appeared some days after inoculation, the disease proceeded without any unusual occurrence. I have not seen this peculiar vesicle in any case but when associated with acute disease.

In page 26, speaking of the peculiar scab of a greyish-brown colour, I have remarked, that it is generally observed in persons of a scorbutic habit;—since that Report I have had an opportunity of seeing it on persons in perfect health;—a pretty good idea may be conceived of it by a reference to Willan's first plate, fig. 3.

To account for this variety in the scab, Dr. W. refers me to an explanation in the Med. and Phys. Journal,* where it is supposed to be altered in its nature by some then existing eruption.—I am sorry I cannot give my support to this explanation; persons affected with what are called herpetic eruptions are recommended to the Infirmary, and are not inoculated until I have succeeded in removing these diseases.

The amber coloured scab is of two kinds,—the first follows the rupture of a vesicle, and cannot be depended on;—the second arises form a ruptured pustule, and differs from the regular scab only in colour.

In the Report of the 9th of January, in the case of Charles Judge, page 257, line 20, *vesicle* by an error of the press is used for *pustule;* a term, as there applied, contradicting the distinction made in the former part of this paper.

In the case of Elizabeth Hall, is not the small-pox to be considered as the constitutional disease, and the cow-pox as only local? I am, &c.

*Nottingham,*

*March* 12, 1806.

JAMES CLARKE,

Physician to the Vaccine Institution.

* Medical and Physical Journal, vol. 14, p. 537.

